# Retraction Note: Ang II-AT2R increases mesenchymal stem cell migration by signaling through the FAK and RhoA/Cdc42 pathways in vitro

**DOI:** 10.1186/s13287-022-02733-2

**Published:** 2022-01-31

**Authors:** Xiu-ping Xu, Hong-li He, Shu-ling Hu, Ji-bin Han, Li-li Huang, Jing-yuan Xu, Jian-feng Xie, Ai-ran Liu, Yi Yang, Hai-bo Qiu

**Affiliations:** 1 Department of Critical Care Medicine, Nanjing Zhongda Hospital, School of Medicine, Southeast University, Nanjing, 210009 People’s Republic of China; 2 Department of Critical Care Medicine, Affiliated Hospital of University of Electronic Science and Technology of China & Sichuan Provincial People’s Hospital, Chengdu, 610072 People’s Republic of China

## Retraction Note: Stem Cell Research & Therapy (2017) 8:164 10.1186/s13287-017-0617-z

The Editors-in-Chief have retracted this article because overlap in cell images was identified in the published Figs. 2, 3 and 6. The authors provided the original files, but the data contained further cases of duplication. The Editor-in-Chief therefore no longer has confidence in the integrity of the data in this article.

All authors agree to this retraction.

